# Case Report: Stress-Induced Childhood-Onset Neurodegeneration With Ataxia-Seizures Syndrome Caused by a Novel Compound Heterozygous Mutation in *ADPRHL2*

**DOI:** 10.3389/fneur.2022.807291

**Published:** 2022-02-11

**Authors:** Aijun Lu, Chunxia Dong, Bihong Chen, Lei Xie, Huaiqiang Hu

**Affiliations:** ^1^Weifang Medical University, Weifang, China; ^2^The 960th Hospital of People's Liberation Army (PLA), Jinan, China

**Keywords:** *ADPRHL2*, ARH3, CONDSIAS, novel phenotypes, histopathology

## Abstract

*ADPRHL2* gene mutations have been demonstrated as the cause of stress-induced childhood-onset neurodegeneration with variable ataxia and seizures (CONDSIAS), an autosomal recessive genetic disorder characterized by an abnormal gait, intellectual disability, seizures, ataxia, other nervous system degenerative diseases, and axonal sensorimotor neuropathy. Since first reported in 2018, ADP-ribosylhydrolase like 2 (*ADPRHL2*) gene mutations in previous cases were all diallelic homozygous. Here, we report a case of CONDSIAS with a novel compound heterozygous mutation in the *ADPRHL2* gene. This patient is presented with autonomic nervous dysfunction manifested as polyuria, gastrointestinal disturbance, and sinus arrhythmia, which may be considered as new clinical manifestations in addition to the above classical manifestations. Muscle biopsy revealed myogenic lesions, which is a previously unreported feature.

## Introduction

The *ADPRHL2* gene encodes the ADP-ribosylated hydrolase protein (ARH3), which is involved in ADP-ribosylation. The ADP-ribosylation is a reversible post-translational modification in which the polypeptide ADP-ribose (PAR) is added to proteins in response to stress. It is negatively regulated by poly ADP- ribosylated hydrolases (PARGs) and by ARH3. Mutations in *ADPRHL2* cause ARH3 dysfunction, thereby leading to abnormal accumulations of PAR in cells. Inactivation of ARH3 leads to increased excitatory toxicity or cell death. Recently, it was observed that the mutations of *ADPRHL2* were related to the stress-induced childhood-onset neurodegeneration with variable ataxia and seizures (CONDSIAS, MIM 618170) (1, 2). All previously reported mutations in this gene have been diallelic homozygous (1–6). The CONDSIAS mainly manifests as neurodevelopmental delay, epilepsy, ataxia, and axonal neuropathy due to stressors such as infection in childhood or adolescence. Patients also exhibit dysarthria, dysmyotonia, facial myoclonus, microcephaly, sensorineural hearing loss, scoliosis, or ocular symptoms (including diplopia, strabismus, nystagmus, and external ophthalmoplegia) (6). Mishra et al. (5) also highlighted the occurrence of longitudinally extensive myelopathy as a novel manifestation of CONDSIAS. Here, we report a novel compound, which is a heterozygous mutation of *ADPRHL2*. This patient is presented with autonomic nervous dysfunction manifested as polyuria, gastrointestinal disturbance, sinus arrhythmia, and myogenic lesions, which may be considered new clinical manifestations of CONDSIAS.

## Case Description

**Figure 1:**
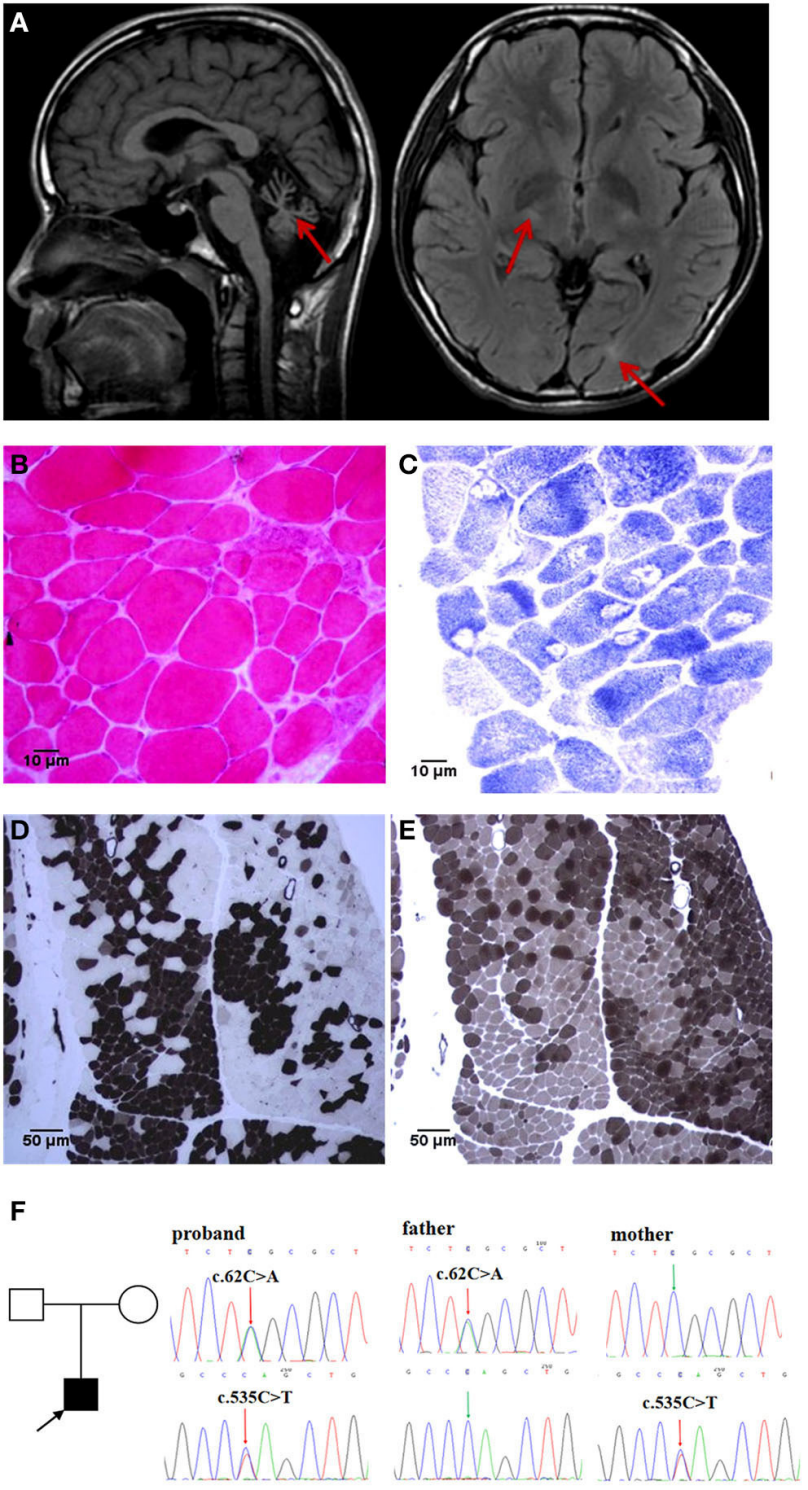
Brain MRI, muscle histopathology, and genetic test findings: **(A)** Brain MRI reveals obvious cerebellar atrophy in T1WI (left), lateral ventricle posterior horn and basal ganglia white matter lesion in FLAIR (right, red arrows). **(B–E)** Skeletal muscle biopsy: HE staining [**(B)**, 40× objective] showed that the muscle fibers were significantly different in size, with atrophied angular fibers and enlarged round fibers. NADH staining [**(C)**, 40× objective] showed obvious atrophy of type I fibers, and more entomophagy occurred in type I than in type II fibers. ATPase staining showed obvious type I and type IIb fiber pathological compensatory hyperplasia, with deep staining spots in the muscle fibers of the histochemistry at pH4.3 [**(D)**, 10× objective] and pH10.4 [**(E)**, 10× objective]. **(F)** WES demonstrated a *de novo* compound heterozygous mutation of *ADPRHL2* in proband, confirmed by Sanger sequencing of asymptomatic parents.

A 15-year-old boy was admitted to a hospital for stumbling and weakness. He had developed cough and diarrhea, followed by an abnormal and unsteady gait without apparent cause at the age of 3. Partial remission was achieved after symptomatic treatment. Since then, he experienced recurrent episodes of ataxia and limb weakness following every incidence of diarrhea. Self-care, learning, and communication abilities declined gradually, which were accompanied by abdominal flatulence, gastrointestinal disturbance, and polyuria. These episodes usually occurred one time or two times a year at first. However, the frequency of such episodes increased, gradually becoming constant at ~3 years after onset. He received treatments with immunoglobulin and glucocorticoid on several occasions, without success or accurate diagnosis of the condition. At the time of admission, he was emaciated, at 150 cm in height and 40 kg in weight. He could not walk independently or sit upright. Physical examination revealed a decreased advanced mental function, dysarthria, right ptosis, nystagmus, hypomyotonia, hypesthesia, and pallhypesthesia in the limbs. The results of the bilateral finger-nose test and heel-knee-tibia test were unstable. He displayed obvious scoliosis and a mild foot drop deformity. The patient was not born prematurely and developed normally prior to the onset of the condition. His parents were not consanguineous. All basic metabolic, cerebrospinal fluid, and microbial pathogen tests were normal. Brain MRI showed marked cerebellar atrophy in addition to signal changes in cerebral white matter (Figure 1A), which was normal up to 8 years prior to the most recent scan. The electromyography showed a fibrillation potential and a positive sharp wave in the left biceps and tibialis anterior muscle. The voltage increased slightly but the polyphase wave increased by more than 40%. The amplitude and conduction velocity of the motor nerve was notably decreased, and the amplitude of the sensory nerve could not lead out. Reflexes of the bilateral tibial nerves were not elicited. These findings revealed neurogenic and myogenic lesions. The neurogenic lesions in question had appeared when the patient was 3 years old. The electroencephalogram showed frequent irregular slow waves with a high amplitude of 1.5–2 Hz in the whole cephalic region. An auditory brainstem response test showed a mild left sensorineural hearing loss. The ECG showed sinus arrhythmia, incomplete right bundle branch block, and electric axis right anteroclock transposition. Upon obtaining from the patient and his parents, a skeletal muscle biopsy was performed. Hematoxylin and eosin staining showed different sizes of muscle fibers. The reduced form of nicotinamide-adenine dinucleotid (NADH) and ATPase-staining showed a pathological aggregation in myofibrillary groups (Figures 1B–E). Whole-exome sequencing was performed on this patient, and Sanger sequencing was applied to verify his parents. A compound heterozygous mutation of *ADPRHL2* was recognized in the proband [NM_017825: c.62C>A, p.[S21^*^]; c.535C>T, and p.[Q179^*^]]. The mutations c.62C>A and c.535C>T were inherited from his father and mother, respectively (Figure 1F).

## Discussion

The ADP ribosylation (ADPr) is a reversible post-translational modification in which PAR is added to proteins in response to stress, involving a variety of physiological and pathological processes, including DNA repair, telomere function, mitotic spindle-formation, intracellular transport, energy metabolism, senescence, and apoptosis (1). In response to stress, PAR levels can be increased by 10–500 times and are rapidly removed by poly ADP-ribose (PAR) glycohydrolase (PARG). Excessive accumulation of PAR-modified protein can trigger a cascade of cell death responses leading to progressive neurodegeneration. Only two known genes have specific PARG activity in humans (*PARG* and *ADPRHL2*); PARG may be a major factor in the removal of PAR under base conditions. The *ADPRHL2* gene (NM_017825, MIM:610624, Gene ID:54936), located at 1p34.3, contains six coding exons that generate a single protein-coding transcript—ARH3, containing 363 amino acids. The ARH3 dysfunction can trigger a cascade of cell death responses leading to progressive neurodegeneration. Studies have found that the accumulation of PAR after *ADPRHL2* mutation is the pathological mechanism that mediates the increase of oxidative stress-induced cell death (2), particularly in neural tissue (7). Recently, *ADPRHL2* gene mutations have been associated with stress-induced CONDSIAS, which is an autosomal recessive genetic disorder characterized by an abnormal gait, intellectual disability, seizures, ataxia, other nervous system degenerative diseases, and axon sensorimotor neuropathy, dysarthria, dystonia, facial myoclonus, microcephaly, sensorineural hearing loss, scoliosis, and eye symptoms (diplopia, strabismus, ophthalmoplegia), induced by stressors in childhood or adolescence (1–3).

In a few reported cases to date, mutations in *ADPRHL2* causing CONDSIAS have all been diallelic homozygous, and in most cases, the parents are inbred (1–6). However, the gene displayed a novel compound heterozygous mutation in this patient. The c.62C>A mutation occurred in exon 1, resulting in the mutation of Serine at position 21 into a stop codon. Another mutation, c.535C >T, occurred in exon 4, mutating glutamine at position 179 into a stop codon, which may lead to premature termination of peptide chain synthesis and further affect protein function (PVS1). This mutation occurs with extremely low frequency in the population (PM2_P). Bioinformatics prediction software, Mutation Taster, predicted that the mutation was likely harmful. This mutation was not included in the ClinVar database. Pedigree verification showed that c.62C>A heterozygous variation was inherited from the father and c.535C>T heterozygous variation was inherited from the mother. Mutations at both sites lead to dysfunction of ARH3, which is the only known enzyme with an activity against the serine-linked mono ADP-ribose (MAR) synthesized by the Poly ADP-ribose transferases PARP1/2-HPF1 complex under stress (8). Deficiency in ARH3 activity results in the persistence of mono ADP-ribose chromatin scars at the sites of single strand break. These scars accumulate and impede local histone acetylation and other histone modifications in ARH3-mutated cells, resulting in a perturbed histone code, deregulated transcription, and cellular dysfunction (9). Additionally, the persistence of chromatin scars in proliferating neural cells, such as glia, may contribute to the relatively early onset of disease in patients who are ARH3-mutated, during pediatric development (2). However, it is likely that other types of stochastic DNA damage trigger ADP-ribose accumulation over longer time periods in post-mitotic neurons, perhaps explaining the degenerative component of this disease.

Among the previously reported cases, the age of onset was between 12 months and 13 years. The onset of symptoms among children of <2 years old showed a developmental delay in early childhood and primarily presented with epilepsy as the first symptom, gradually presenting speech and motor disorders with progressive aggravation; however, the stressor could not be identified (1). In older children, the first symptoms were gait abnormalities and ataxia following identifiable stress, such as infection, surgery, or exercise. Epilepsy is associated with approximately two-thirds of the patients and is likely to be a risk factor for poor prognosis and death. Those with later onset CONDSIAS, aged 10–13 years, present only with neurological degeneration, usually without epilepsy, and have a good prognosis (6). In the adult patients reported by Durmus (3), most have abnormal behavior as a core symptom, and the condition manifests as episodic psychosis, ataxia, and motor neuropathy with pyramidal signs, known as PAMP syndrome.

In addition to prominent neurodegeneration and ataxia, axonal sensorimotor neuropathy, autonomic dysfunction such as ventosity, gastrointestinal intolerance, frequent urination, and sinus arrhythmia are likely to be the clinical phenotype of this new complex heterozygous mutation, having no other explanatory causes following a clinical examination. Low activation of *ADPRHL2* and high activation of poly-ADP-Ribose polymerase 1, leading to excessive accumulation of PAR, have been reported to be closely associated with gastrointestinal symptoms (10). Therefore, in cases where symptoms always appear and worsen after diarrhea, episodic diarrhea is likely to be a manifestation of this clinical syndrome, which also acts as a stress factor with fever. Approximately, one-third of the reported patients died from acute cardiac arrest, and Mishra et al. described the neurogenic cardiac arrest as a possible consequence of the disease (5), although there has not been any report of cardiac abnormalities to date. Whether such mutations are related to autonomic nerve damage requires further investigation, but it provides an important suggestion that changes to the heart may occur early in the disease. Brain MRI primarily showed cerebellar atrophy, especially vermis atrophy (2). In this case, the white matter signals were of interest. Four patients were reported to have muscle biopsies showing neurogenic changes (2–5), the nerve biopsies showed a severe axonal loss in two cases (2–4). In this study, electrophysiology and muscle biopsy both confirmed the presence of myogenic damage, and the apparent atrophy of type I myofibrils accompanied by the phenomenon of insect eating, which was significantly different from the findings of previous reports, and, thus, is another novelty of the study.

The novel pathogenic mutation of *ADPRHL2* in CONDSIAS expands the genetic spectrum of the condition. At present, there is no clear relationship between clinical phenotype and gene mutation, and electrophysiology and pathology need to be further studied in the future.

## Patient Perspective

During our follow-up visit, after 2 years of energy medication and self-training, his symptoms of gait instability and learning ability had gradually improved, for which his parents were very grateful.

## Data Availability Statement

The datasets presented in this article are not readily available due to ethical and privacy restrictions. Requests to access the datasets should be directed to the corresponding author.

## Ethics Statement

The study involving human participants was reviewed and approved by the Ethics Review Committee, the 960th Hospital of PLA. Written informed consent to participate in this study was provided by the participants' legal guardian/next of kin. Written informed consent was obtained from the individual(s), and minor(s)' legal guardian/next of kin, for the publication of any potentially identifiable images or data included in this article.

## Author Contributions

AL and CD contributed equally in drafting the manuscript and ensuring the accuracy of medical content. BC and LX interpreted the data and revised the manuscript for intellectual content. HH revised the manuscript for content and ensured the accuracy of medical content. All authors contributed to the article and approved the submitted version.

## Conflict of Interest

The authors declare that the research was conducted in the absence of any commercial or financial relationships that could be construed as a potential conflict of interest.

## Publisher's Note

All claims expressed in this article are solely those of the authors and do not necessarily represent those of their affiliated organizations, or those of the publisher, the editors and the reviewers. Any product that may be evaluated in this article, or claim that may be made by its manufacturer, is not guaranteed or endorsed by the publisher.
